# Discovery of Hordenine as a Potential Inhibitor of Pyruvate Dehydrogenase Kinase 3: Implication in Lung Cancer Therapy

**DOI:** 10.3390/biomedicines8050119

**Published:** 2020-05-14

**Authors:** Saleha Anwar, Taj Mohammad, Anas Shamsi, Aarfa Queen, Shahnaz Parveen, Suaib Luqman, Gulam Mustafa Hasan, Khalid A. Alamry, Naved Azum, Abdullah M. Asiri, Md. Imtaiyaz Hassan

**Affiliations:** 1 Centre for Interdisciplinary Research in Basic Sciences, Jamia Millia Islamia, Jamia Nagar, New Delhi 110025, India; email2saleha@gmail.com (S.A.); taj144796@st.jmi.ac.in (T.M.); mshamsi1@jmi.ac.in (A.S.); 2 Department of Chemistry, Jamia Millia Islamia, Jamia Nagar, New Delhi 110025, India; aarfa30@gmail.com; 3 Molecular Bioprospection Department, CSIR-Central Institute of Medicinal and Aromatic Plants, Lucknow 226015, India; shahnazparveen4@gmail.com (S.P.); s.luqman@cimap.res.in (S.L.); 4 Academy of Scientific and Innovative Research (AcSIR), Ghaziabad 201002, India; 5 Department of Biochemistry, College of Medicine, Prince Sattam Bin Abdulaziz University, P.O. Box 173, Al-Kharj 11942, Saudi Arabia; mgulam@gmail.com; 6 Chemistry Department, Faculty of Science, King Abdulaziz University, P.O. Box 80203, Jeddah 21589, Saudi Arabia; kalamri@yahoo.com (K.A.A.); navedazum@gmail.com (N.A.); asiri2@gmail.com (A.M.A.); 7 Center of Excellence for Advanced Materials Research (CEAMR), King Abdulaziz University, P.O. Box 80203, Jeddah 21589, Saudi Arabia

**Keywords:** pyruvate dehydrogenase kinase, kinase inhibitors, drug design and discovery, lung cancer therapy, molecular dynamics simulation, hordenine

## Abstract

Design and development of potential pyruvate dehydrogenase kinase 3 (PDK3) inhibitors have gained attention because of their possible therapeutic uses in lung cancer therapy. In the present study, the binding affinity of naturally occurring alkaloids, hordenine, vincamine, tryptamine, cinchonine, and colcemid was measured with PDK3. The molecular docking and fluorescence binding studies suggested that all these compounds show a considerable binding affinity for PDK3. Among them, the affinity of hordenine to the PDK3 was excellent (*K* = 10^6^ M^−1^) which was further complemented by isothermal titration calorimetric measurements. Hordenine binds in the active site pocket of PDK3 and forms a significant number of non-covalent interactions with functionally important residues. All-atom molecular dynamics (MD) simulation study suggested that the PDK3-hordenine complex is stabilized throughout the trajectory of 100ns and leads to fewer conformational changes. The enzyme inhibition studies showed that hordenine inhibits the activity of PDK3 with an IC_50_ value of 5.4 µM. Furthermore, hordenine showed a cytotoxic effect on human lung cancer cells (A549 and H1299) with an admirable IC_50_ value. However, it did not inhibit the growth of HEK293 cells up to 200 µM, indicating its non-toxicity to non-cancerous cell lines. In summary, our findings provide the basis for the therapeutic implication of hordenine and its derivatives in lung cancer and PDK3-related diseases after required in vivo validation.

## 1. Introduction

Inhibition of protein kinase is an exciting domain of drug design and discovery especially in the context of cancer and neurodegenerative diseases because protein kinases are amongst the important drug targets [1]. More than 80% of cellular pathways are governed by protein kinases because protein phosphorylation and dephosphorylation regulate many cellular pathways, and any disruption in their function is often associated with the development of diseases [2]. Protein kinases are exploited as potential drug targets for different types of cancers [3], metabolic disorders [4], and neurodegenerative diseases [5]. FDA approved inhibitors in different phases of clinical trials have already been developed for cyclin-dependent kinase, aurora kinases, mTOR, Tyrosine kinases, MAP kinases, and many others [6]. There are many kinases, which play a significant role in cellular signaling, and their overexpression is a leading cause of carcinogenesis and metastasis [7].

Pyruvate dehydrogenase complex (PDC) is involved in the maintenance of glucose homeostasis in mammals. It connects two major pathways of ATP generation, glycolysis, and Krebs cycle. The PDC’s complex is regulated through systematic phosphorylation by kinases and dephosphorylation by phosphatases in a well-coordinated manner [8]. Pyruvate dehydrogenase kinase (PDK) isoforms (PDK 1–4) regulate the function of PDC through reversible phosphorylation at specific serine residue in the E1α subunit of PDC which inhibits the function of PDC complex [9]. Amongst all isoforms, the activity of PDK3 is determined by its robust binding to the L2 domain [10] and its overexpression is related to the different types of cancers [11]. In cancer cells, metabolism is altered, and the glycolysis process is subsequently modulated t irrespective of the presence of oxygen. This phenomenon, commonly referred to as the Warburg effect [12], is a hallmark for the metabolic switch in cancer which is associated with the production of enough energy to help the survival of the cancer cells in the presence of limited resources. The normal cells derive most of their metabolic needs from oxidative phosphorylation (OXPHOS) while the cancer cells primarily depend on glycolysis to meet most of their requirements. The inactivation of PDC which in turn depends on the cooperative action of PDKs resulted in aerobic glycolysis and OXPHOS loss which is implicated in many cancers [13].

PDK3 transcription is known to be upregulated in the presence of HIF-1α, which results in mitochondrial respiration inhibition and a dramatic metabolic shift in the dependency of the cell on cytoplasmic glycolysis [14]. The conversion of pyruvate to acetyl-CoA is catalyzed by PDC. The pyruvate affects the activities of PDK1, 2, and 4 but importantly feedback inhibition does not have any effect on PDK3 activity [10]. The unique trait of PDK3 suggests its critical importance in the metabolic switch. Thus, PDK3 plays a key role in metabolic switch control during the progression of cancer and is implicated in cell survival coupled with hypoxia-induced metabolic switch [15]. The positive correlation between overexpression of PDK3 and metastatic progression suggests the importance of PDK3 as a drug target in cancer therapeutics [16,17].

Cancer is amongst the leading causes of death across the globe with a rapid decrease in the survival rate despite all the advances in medical researches. One of the major causes of failure in chemotherapy is multidrug resistance [18]. In the last few decades, there has been an increase in the search for plant-derived drugs for cancer therapy because they have the least side effects and have potential therapeutic properties [19,20,21,22]. Medicinal plants have been used from ancient times to cure various diseases including cancer. The enormous structural and chemical diversity of the natural compounds enables them to be used as an excellent platform in search of drugs that can be used in anticancer therapy [23]. Natural compounds such as polyphenols, alkaloids, and others have been established as potential anticancer agents. Alkaloids have been traditional molecules of interest due to their pronounced physiological activities [24,25,26]. Many alkaloids are used as chemotherapeutic drugs for the treatment of a variety of cancers [27].

Hordenine is an alkaloid isolated from the marine algae, belonging to the phenylethylamine group, having numerous health benefits [28]. Hordenine is a nootropic (non-pharmaceutical cognitive enhancers) compound that augments cognitive ability [29]. It is an MAO-B inhibitor that increases the norepinephrine level and hence, considered as norepinephrine and noradrenaline uptake inhibitor [30]. The systolic and diastolic pressure increased upon hordenine treatment coupled with an increase in peripheral blood volume [31]. There was an observed inhibition of gut movement but interestingly no effect was seen on the psychometrical behavior of mice. The biological activity of hordenine has not been investigated to a great extent. This is for the first time we report inhibition of kinase activity and the consequential anticancer potential of hordenine.

In the present study, we have screened a series of alkaloids to investigate their inhibitory potential towards PDK3. The molecular docking was performed to investigate their binding pattern and the strength of interactions. We further assessed the structural flexibility and dynamic stability of PDK3 in the presence of hordenine by utilizing molecular dynamics (MD) simulations for 100ns. In silico studies were further validated by fluorescence binding and isothermal titration calorimeter (ITC) measurements, suggested an appreciable binding affinity of hordenine to PDK3. Treatment of human lung cancer cells with hordenine significantly inhibits their viability with an admirable IC_50_ value.

## 2. Materials and Methods

### 2.1. Materials

The bacterial culture medium, Difco LB broth Miller (Luria−Bertani), was obtained from Becton, Dickinson, and Company (Sparks, MD, USA). The Ni-NTA resin and gel-filtration column (Superdex-75) were obtained from GE Healthcare (GE Healthcare Life Sciences, USA). All other chemicals used for buffer preparation were analytical grade and obtained from SRL Chemicals (India). Dulbecco’s modified eagle’s media (DMEM), RPMI-1640 and F-12K cell culture medium, antibiotic antimycotic cocktail (penicillin, streptomycin, and amphotericin-B), fetal bovine serum (FBS), MTT (3-[4,5-dimethylthiazol-2-yl]-2,5-diphenyltetrazolium bromide) and cell detachment enzyme (TrypLE express) were purchased from Gibco-life technologies, Thermo Fisher Scientific (USA). Hordenine, vincamine, tryptamine, cinchonine, and colcemid were purchased from Sigma-Aldrich Chemical Co. Bengaluru, India (now Merck KGaA, Darmstadt, Germany). Human adenocarcinoma alveolar basal epithelial cells (A549), lung metastatic cell line (H1299), and human embryonic kidney cells (HEK293) were obtained from the National Centre for Cell Sciences, Pune, India.

### 2.2. Expression and Purification of PDK3

PDK3 was cloned, expressed, and purified by following our published protocol [32]. The purity of PDK3 was checked using sodium dodecyl sulfate-polyacrylamide gel electrophoresis (SDS-PAGE) and confirmed with a single band.

### 2.3. Fluorescence Spectroscopy

The binding affinity of ligands to PDK3 was analyzed by monitoring changes in the fluorescence emission intensity of protein [33]. Fluorescence measurements were carried out on the Jasco spectrofluorimeter (FP-6200, Japan). The experimental parameters were, *λ*_exc_ as 280 nm and *λ*_em_ in the range of 300–400 nm with slit width: 10 nm and medium sensitivity. All the measurements were carried out in triplicates and blank subtracted spectra were reported. The observed fluorescence values were corrected for the inner filter effect [34]. Protein was titrated with an increasing concentration of ligands and each titration of protein. A decrease in the fluorescence intensity was mathematically evaluated using the modified Stern-Volmer equation [35] to estimate the binding parameters for hordenine-PDK3 interaction.

### 2.4. Enzyme Inhibition Assay

Following our published protocols, an enzyme inhibition assay was carried out [36]. Freshly prepared ATP (200 µM) was added to PDK3 (4 µM) and final reaction mixture of 100 μL was incubated at 25 °C for 1 h. Malachite green (200 μL) was further added to the reaction mixture to stop the reaction followed by incubation of samples at room temperature for 20–25 min for the development of color. From the final reaction mixture, 100 μL was transferred to a 96-well plate in triplicate to measure spectrophotometrically at 620 nm.

### 2.5. Isothermal Titration Calorimetry

ITC measurements were carried out at 25 °C on a VP-ITC microcalorimeter from MicroCal, Inc (GE, MicroCal, Northampton, MA, USA). The purified protein was extensively dialyzed and degassed before use to avoid the bubble problem. DMSO was added in an equal amount to the protein solution (1% *v*/*v*) to prevent signal stability problems during ITC measurements. The first injection was a false one (2 μL) and then successive injections of 10 μL followed by the initial false injection. The sample cell contained PDK3 while the syringe was filled with the hordenine. Data analysis was carried out using the attached MicroCal Origin 8.0. The values (*K*_a_, Δ*H*, Δ*S*) were calculated after curve fitting as described in our previous communications [37,38].

### 2.6. Molecular Docking

Atomic coordinates of human PDK3 were taken from the Protein Data Bank (PDB ID: 1Y8O) and heteroatoms were removed [39]. To the polar groups in the protein, hydrogens were added coupled with the Kollman charges using MGL tools [40]. The docking was performed using Auto Dock Vina which was structurally blind with exhaustiveness of 8. The binding affinity and interaction of hordenine towards PDK3 was analyzed using PyMOL and Discovery Studio. The detail of the docking method has been described elsewhere [41,42].

### 2.7. MD Simulations

All-atom MD simulations were performed on PDK3 before and after hordenine binding for 100 ns at 300 K at the molecular mechanics level using GROMOS 54A7 force-field in GROMACS 5.1.2 [43]. The PRODRG server was used to generate topology parameters for hordenine and fused into the PDK3 topology to generate a protein-ligand complex system. Further, PDK3 and PDK3-hordenine systems were solvated in a cubic box with the Simple Point Charge (spc216) model to simulate them in the aqueous environment. The energy minimization using the 1500 steps of the steepest descent method was performed for both the systems. The equilibration was carried out for 100 ps at constant volume under periodic boundary conditions with a stable environment of 1 bar pressure. The final MD run was performed for 100,000 ps for both systems where trajectory for PDK3 apo was taken from our previous study [44]. The inbuilt utilities of GROMACS were used to analyze both trajectories, and VMD [45] and QtGrace were used for visualization purposes and plotting the graph. Detailed information about the MD simulations has been reported previously [46,47,48].

### 2.8. Cell Culture and Cytotoxicity Studies

Cell cultures of H1299 and A549 cells were respectively maintained in RPMI-1640 and F12K cell growth medium, while HEK293 cells were maintained in DMEM (having 10% heat-inactivated FBS and 1% antibiotic-antimycotic solution) in a humidified CO_2_ incubator (5% CO_2_, 37 °C). These lung cancer cell lines were selected based on previous reports related to PDK3 [49]. Hordenine was evaluated for cell growth inhibition potential using the MTT assay as described [50,51]. Briefly, the selected cells were plated in a 96-well culture plate (6000–7000 cells/well), and after 24 h cells were incubated with hordenine (0–200 μM) for 48 h. Following 48 h incubation of cells with hordenine, 25 µL MTT (from 5 mg/mL stock) was added and incubated for 4–5 h at 37 °C in a CO_2_ incubator. The resultant formazan was dissolved in 100 µL of DMSO and the absorbance was measured at 570 nm using a multiplate ELISA reader (BioRad). The percentage of cell viabilities was estimated using absorbance data and plotted as a function of hordenine concentration. Respective DMSO treatment was taken as vehicle control and subtracted from corresponding hordenine treatment groups, whereas for anticancer studies paclitaxel has been taken as a positive control.

### 2.9. Statistical Analysis

All the experiments were performed in triplicate and the data obtained has been expressed as mean ± standard error of the mean (SEM).

## 3. Results and Discussion

### 3.1. Screening of Natural Compounds with PDK3

Molecular docking and fluorescence spectroscopy were used to screen a series of naturally occurring alkaloids namely hordenine, vincamine, tryptamine, cinchonine, and colcemid against PDK3 (Supplementary Table S1). Finally, hordenine was selected based on the binding affinities calculated from the docking analysis and fluorescence spectroscopy along with the interactions with the functionally important residues of PDK3.

### 3.2. Molecular Docking

**Figure 1:**
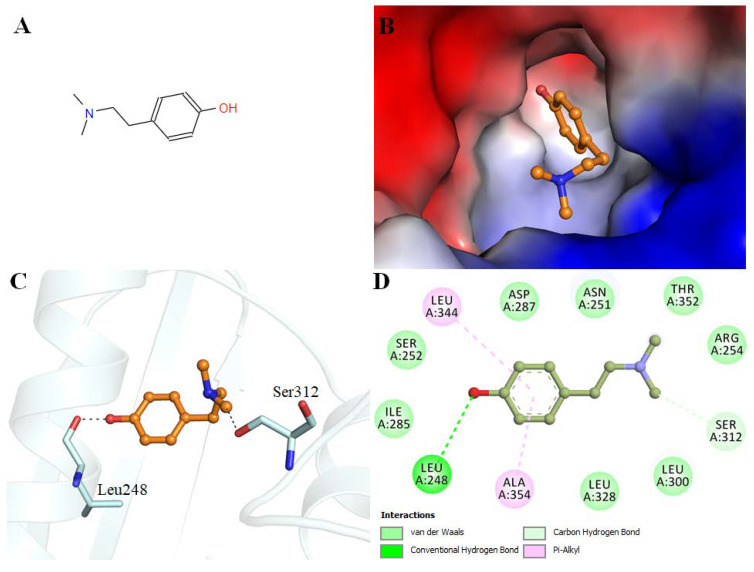
Interactions of hordenine with PDK3. (**A**) 2D representation of hordenine structure. (**B**) Surface view of PDK3 binding pocket occupied by hordenine. (**C**) Hordenine interacting with binding site residues of PDK3 is depicted in cartoon representation. (**D**) 2D structural representation of PDK3 residues interacting with hordenine.

Hordenine shows an appreciable binding affinity to the PDK3 (−7.1 kcal/mol). Interaction analysis of the docked conformers of hordenine was carried out to investigate its possible interactions with the residues of the active site of PDK3. Hordenine interacts with the functionally important residues of PDK3. The ATP binding site Asp287 participates in PDK3-hordenine interactions. The chemical structure of hordenine and its binding pattern with PDK3 is illustrated in Figure 1.

### 3.3. Conformational Dynamics Calculation

**Figure 2:**
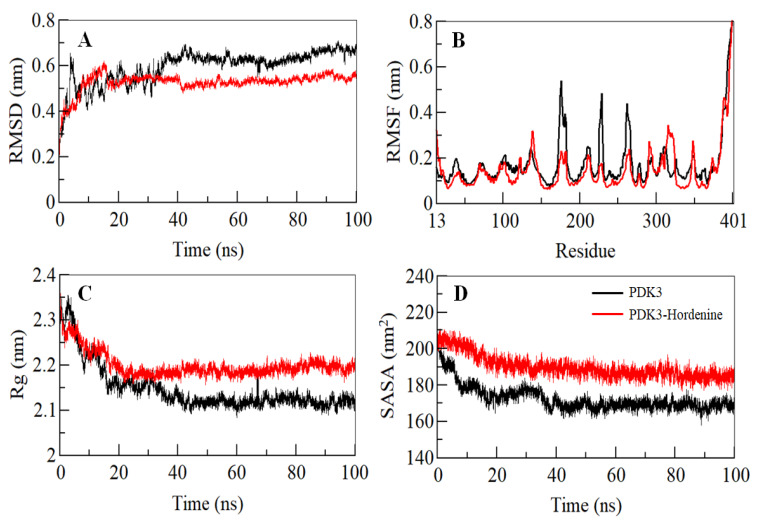
Structural dynamics of PDK3 as a function of time. (**A**) RMSD plot of PDK3 prior and post hordenine binding. (**B**) Residual fluctuations plot of free PDK3 and PDK3-hordenine complex. (**C**) Time evolution of the radius of gyration. (**D**) SASA plot of PDK3 as a function of time. The values were obtained after carrying out the 100 ns MD simulation study. The black corresponds to PDK3 apo while red shows values obtained for and PDK3-Hordenine complex.

Binding of a small molecule can lead to conformational alterations in a protein. Root-mean-square deviation (RMSD) can give clues about the structural changes and the dynamic behavior of a protein [52]. The average RMSD for PDK3 before and after hordenine binding was calculated and found to be 0.59 nm and 0.53 nm, respectively. A decreased RMSD of PDK3 in the presence of hordenine suggests a stabilization of PDK3 in the presence of hordenine as compared to the free PDK3 (Figure 2A). The RMSD of PDK3 in the presence of hordenine suggests strong stability of the PDK3-hordenine complex evident from an apparent equilibration with no switching throughout the trajectory (Figure 2A).

To explore the residual fluctuations in PDK3 and PDK3-hordenine complex, the average fluctuation of each residue was estimated as the root-mean-square fluctuation (RMSF) shown in Figure 2B. The average RMSF for PDK3 and PDK3-hordenine complex was calculated as 0.17 nm and 0.15 nm, respectively. It was observed that random residual fluctuations in PDK3 were minimized on hordenine binding. The investigation of the RMSF plot suggested the least residual fluctuation in the regions where hordenine binds. A decreased fluctuation in PDK3 after hordenine binding suggested the strong stability of the complex.

The radius of gyration (*R_g_*) gives an insight into the compactness and folding behavior of a protein [53]. *R_g_* values were computed for both the systems and the average *R_g_* for PDK3 and PDK3-hordenine complex was calculated as 2.14 nm and 2.19 nm, respectively. The *R_g_* plot signifies that the magnitude of *R_g_* value increases slightly after binding of hordenine and this increase can be owed to its packing. No switching was observed in the *R_g_* of PDK3 in the presence of hordenine, and it attains a stable equilibrium thus signifying the stability of the complex throughout the trajectory (Figure 2C).

The solvent-accessible surface area is the interface between a protein and its surrounding solvent and serves as a parameter that can study the conformational dynamics in a protein under solvent conditions [54,55]. The calculated SASA of PDK3 and PDK3-hordenine complex systems provided an insight into their conformational behavior during the simulation. The average SASA for PDK3 and PDK3-Hordenine complex was calculated as 172.64 nm^2^, and 188.42 nm^2^, respectively. There was a slight increment in the SASA value of the PDK3-hordenine system and this increase is attributable to the increased surface area of PDK3 in presence of hordenine as some inner residues might be exposed to the surface (Figure 2D). The SASA attained a stable equilibrium without any switching thus implying the structural stability of PDK3 in the presence of hordenine.

### 3.4. Hydrogen Bond Analysis

**Figure 3:**
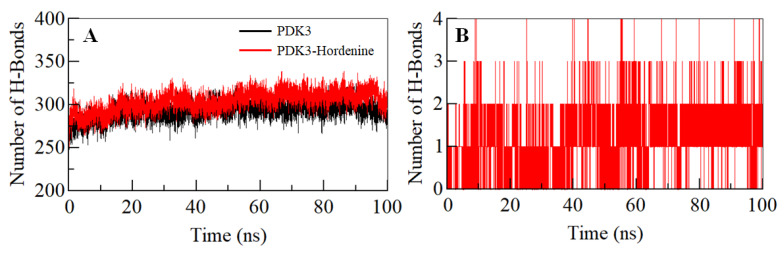
Time evolution and stability of hydrogen bonds. (**A**) Intramolecular within PDK3, and (**B**) intermolecular between Hordenine and PDK3.

The intramolecular hydrogen bonds (H-bonds) in proteins play a pivotal role in defining their stability and can be utilized to investigate the stability of the protein-ligand complex [56,57]. To validate the stability of the PDK3 and PDK3-hordenine docked complex, we have computed the dynamics of intramolecular H-bonds paired within 0.35 nm. The average number of intramolecular H-bonds in PDK3 before and after hordenine binding was found to be 293 and 301, respectively (Figure 3A). There was an increase in hydrogen bonding within PDK3 suggesting a decrease in the dynamics post binding of hordenine. Further, the dynamics of intermolecular H-bonds were analyzed between hordenine and PDK3 paired within 0.35 nm to investigate the complex stability. There are 1–2 intermolecular H-bonds shared by hordenine and PDK3 which are consistent throughout the simulation trajectory (Figure 3B). All these observations suggest the binding of hordenine in the active pocket of PDK3 with 1–2 H-bonds with stability and up to 3–4 H-bonds with higher fluctuation which is per our molecular docking observations.

### 3.5. Fluorescence-Based Binding Studies

**Figure 4:**
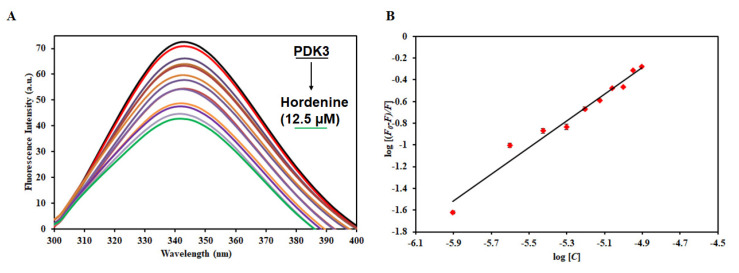
Binding studies of hordenine with PDK3. (**A**) Fluorescence emission spectra of PDK3 (4 μM) with the increasing concentration of hordenine (0–12.5 µM). (**B**) Modified Stern−Volmer plot obtained from the quenching of PDK3 fluorescence with increasing concentration of hordenine.

To measure the actual binding affinity of hordenine to PDK3, fluorescence binding studies were performed as described [58]. PDK3 shows an emission maximum of around 344 nm, a characteristic of a native protein. We observed a decrease in the fluorescence intensity with increasing concentration of hordenine (Figure 4). This decrease in fluorescence intensity of PDK3 in the presence of hordenine suggests the formation of a complex between PDK3 and hordenine [59]. The decrease in fluorescence intensity was mathematically analyzed using a double log relation (modified Stern−Volmer equation) to find the value of binding constant (*K*). The value of the binding constant (*K*) for the hordenine was estimated as 0.5 × 10^6^ M^−1^, indicating an excellent affinity to PDK3.

### 3.6. Enzyme Inhibition Assay

**Figure 5:**
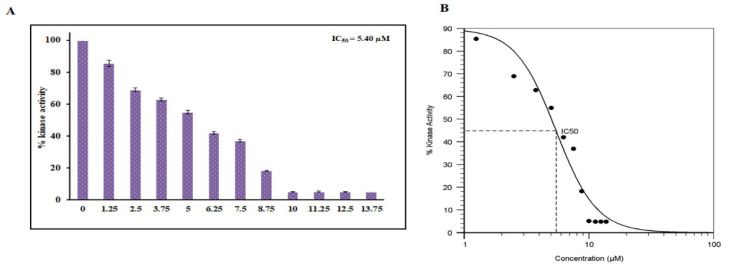
Enzyme inhibition studies of PDK3 with hordenine. (**A**) ATPase inhibition assay of PDK3 with increasing concentration of RA (0–13.75 µM). The activity of native PDK3 was taken as 100% for reference. (**B**) Calculation of IC_50_ employing AAT Bioquest calculator.

To further validate our fluorescence-based binding studies, an enzyme inhibition assay was carried out with increasing concentrations of hordenine to check the kinase inhibitory potential. The kinase activity was quantified and plotted as percent inhibition. The kinase activity of PDK3 alone was taken as 100% for reference. It is clear from Figure 5 that with an increasing concentration of hordenine (0–13.75 µM), there was a dose-dependent decrease in PDK3 activity. IC_50_ is the concentration of a drug/ligand at which it shows 50% of its inhibitory effect. AAT Bioquest calculator was used to measure IC_50_ of hordenine and it was found 5.4 µM. The results of the ATPase assay suggest hordenine is a potent inhibitor of PDK3.

### 3.7. Isothermal Titration Calorimetry

**Figure 6:**
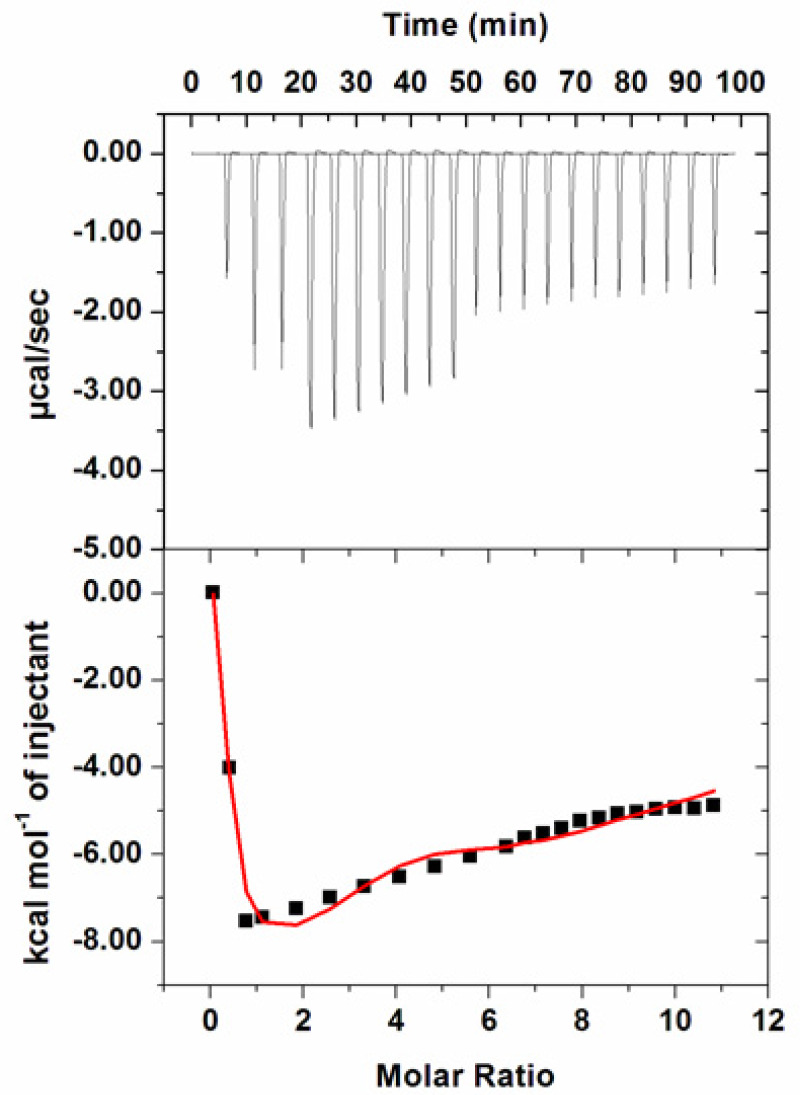
Isothermal titration calorimetric measurement of hordenine titration with PDK3. (Top) Raw data plot of heat produced against time for the titration of 500 µM hordenine into 15 µM PDK3. The lower panel shows the binding isotherm obtained after the integration of peak area and normalization to yield a plot of molar enthalpy change against the hordenine/PDK3 ratio. The fit curve is shown in the red color line obtained after four model sites.

Thermodynamic parameters associated with the interaction of hordenine to PDK3 were measured by ITC as described [35]. During fluorescence binding studies hordenine showed a strong binding affinity and reduced enzyme activity of PDK3 to a greater extent. Figure 6 shows a typical isotherm obtained after titrating 500 µM hordenine with 15 µM PDK3. Various studies report different values of thermodynamic parameters obtained from fluorescence spectroscopy and ITC and this is because ITC measures a global change in the thermodynamic properties while fluorescence spectroscopy takes into account only the local changes around the fluorophore (Trp214) [60]. In the upper panel, raw data with negative heat pulses is shown which confirms exothermic binding. Binding curves found after subtraction of dilution heat of both ligands and protein are depicted in the bottom section. The obtained ITC isotherm advocates the spontaneous binding of hordenine with PDK3. The results presented were obtained from four site model fitting and thermodynamic parameters obtained for this interaction are presented in Table 1.

### 3.8. Cell Culture and Viability Studies

**Figure 7:**
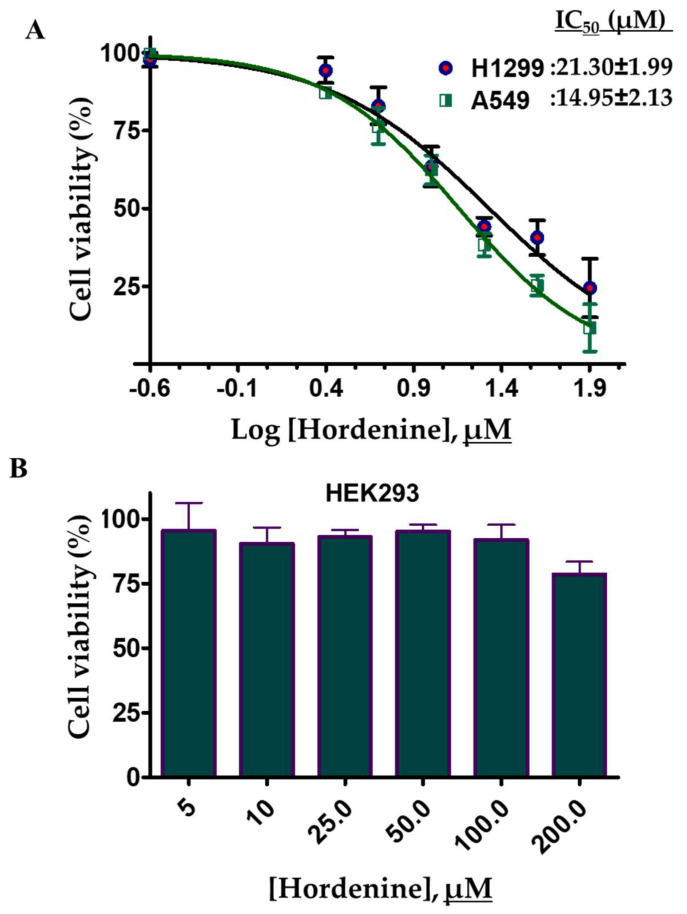
Cell viability studies of hordenine with selected lung cancer cell lines and HEK293 cells: Lung cancer cell lines (H1299 and A549) and HEK293 cells were treated with increasing concentrations of hordenine and cell viabilities were measured using MTT assay. (**A**) Cell growth inhibition curve of hordenine on H1299 and A549 cells. (**B**) Cell cytotoxicity studies of HEK293 cells assessed with different concentrations of hordenine. Percent of cell viabilities was estimated for DMSO treated vehicle control cells. Each data point shows the mean ± SD from n = 3. Note, the x-axis denotes the percentage of cell viability.

Hordenine binds strongly to PDK3 and decreases its kinase activity. PDK3 is an important enzyme associated with growth and cell cycle regulation of different cells [11,61]. Overexpression of PDK3 helps in the growth of different cancer cells [49]. Thus, we have evaluated the effect of hordenine treatment on the cell viability of human lung cancer (A549 and H1299) cells. To access the cell viability of selected cancer cells, the cells were treated with increasing concentrations of hordenine and cell viabilities were determined using the MTT assay. The results of cell viability studies showed that hordenine inhibited the growth of A549 and H1299 cells in a dose-dependent mode (Figure 7A). Using a growth inhibition curve the estimated IC_50_ values of hordenine for A549 and H1299 cells were 14.95 ± 2.13μM and 21.30 ± 1.99μM, respectively. The cytotoxicity or cell viability studies of hordenine were also evaluated on HEK293 cells and found that in the studied concentration range (0–200 μM), it did not inhibit the growth of HEK293 cells (Figure 7B). These results suggest that hordenine decreases the viability of selected human lung cancer cell lines as compared to the vehicle control (DMSO) and thus can be evaluated further for the development of potential lead molecules for the targeting of PDK3 or associated cancers. However, the PDK3 inhibition assay was not conducted for the cancer cell lines.

## 4. Discussion

Most of the human cancers are directly or indirectly linked to the signaling pathways and activations of these pathways often play a vital role in cancer development and progression [62,63]. Protein kinases are at the heart of these signaling cascades and hence, are the major attraction for researchers to be utilized as drug targets [64]. In cancer cells, the source of metabolism is aerobic glycolysis, and hence swapping from the oxidative phosphorylation to aerobic glycolysis is a major trait of cancer cells that enables cancer cells to survive and obtain energy. PDK3 plays a pivotal role in this metabolic switching during the progression of cancer and is involved in cell survival and hypoxia-induced metabolic alterations thus highlighting its importance as a prominent target for cancer therapy [15]. Many studies report overexpression of PDK3 in many cancers and a positive relationship of PDK3 expression was noticed with the cancer progression [11]. All these studies validate PDK3 as a potential drug target for cancer therapy and support the development of therapeutic molecules against PDK3 [44].

One of the major goals in structure-based drug discovery is that designed drugs must be selectively toxic to only cancer cells with no cytotoxicity towards normal cells [65]. The use of phytochemicals or natural products to treat human diseases dates to ancient times due to broad-spectrum properties possessed by them [66,67]. The most important aspect associated with them is their diversity and minimal side effects. These compounds have been used as leads for drug discovery to treat neurodegenerative diseases, cancers, and other diseases. In this study, a series of alkaloids was screened against PDK3 to check the inhibitory potential of these natural compounds.

Initially, molecular docking and fluorescence spectroscopy were used to screen the best compound. Amongst all, hordenine was found to be the best inhibitor of PDK3 and was taken further for the detailed analysis. Thus, molecular docking and MD simulation studies provided atomistic details of PDK3-hordenine interaction to provide an insight into the mechanism of inhibition. The molecular docking analysis suggested the binding of hordenine with PDK3 with a detailed pattern of binding and interactions. We found that hordenine interacts with the functionally important residues of PDK3 (Figure 1). To see an atomistic detail of the binding mechanism of PDK3 and hordenine, a 100 ns MD simulation was performed. An efficient binding pattern was observed between PDK3 and hordenine, and a stable complex is formed with minimal structural changes over the simulation time. The magnitude of RMSD, RMSF, *R*_g,_ and SASA implies the stabilization of the PDK3 structure by hordenine without any considerable conformational change (Figure 2).

In silico observations were further validated by in vitro studies. The fluorescence measurements suggested a significant binding between PDK3 and hordenine with a binding constant of 10^6^ M^−1^. ITC further advocated the spontaneous nature of binding evidence from the obtained isotherm. The cell-free enzyme inhibition assay suggested inhibition of PDK3 by hordenine with an IC_50_ of 5.4 µM and hence, was proved as an effective inhibitor of PDK3. Further, hordenine was evaluated against human lung cancer cell lines and found to inhibit the growth of lung cancer cells. The results of this study are in agreement with the previous studies, which also suggested that inhibition of PDK3 using synthetic or natural molecules leads to a decrease in cancer cell growth [68,69,70]. Finally, our study suggested that hordenine is a potential inhibitor of PDK3 with a promising anti-cancer use. Hence, hordenine itself or its derivatives may be exploited in the development of potent and selective PDK3 inhibitors for the clinical management of cancer and other PDK3 associated diseases.

## 5. Conclusions

Targeting PDK3 by developing new therapeutic molecules appears to be an appealing approach in anticancer therapy. There are severe side-effects associated with chemically synthetic molecules, which encourage the use of phytochemicals as lead molecules in anticancer therapy. Thus, targeting PDK3 by these natural compounds can be an attractive therapeutic strategy to identify and develop small molecule inhibitors. We have established that hordenine may be exploited as a novel scaffold to inhibit the kinase activity of PDK3 along with a considerable cytotoxic effect on lung cancer cell lines. All these observations suggested the implication of hordenine in the therapeutic management of lung cancer and other PDK3 associated diseases.

## Figures and Tables

**Table 1:** Thermodynamic parameters obtained from ITC measurements.

*K*_a_ (Association Constant), M^−1^	∆*H* (Enthalpy Change), cal/mol	∆*S* (cal/mol/deg)
*K_a1_ = 1.95 × 10^4^ ± 2.4 × 10^3^*	*∆H_1_ = 4151 ± 1.2 × 10^3^*	*∆S_1_ = 33.5*
*K_a2_ = 9.3 × 10^4^ ± 7.4 × 10^3^*	*∆H_2_ = −5.11 × 10^4^ ± 4.18 × 10^3^*	*∆S_2_ = −149*
*K_a3_ = 5.1 × 10^4^ ± 3.5 × 10^3^*	*∆H_3_ = 3.75 × 10^4^ ± 5.20 × 10^3^*	*∆S_3_ = 147*
*K_a3_ = 2.5 × 10^3^ ± 1.7 × 10^2^*	*∆H_4_ = −2.33 × 10^5^ ± 1.03 × 10^4^*	*∆S_4_ = −767*

## Supplementary Materials

The following are available online at https://www.mdpi.com/2227-9059/8/5/119/s1, Table S1. Binding parameters of screened natural compounds with PDK3 obtained from molecular docking and fluorescence binding studies.
