# Supplementary material for: Discovery of Hordenine as a Potential Inhibitor of Pyruvate Dehydrogenase Kinase 3: Implication in Lung Cancer Therapy
Source: Biomedicines. 2020 May 14;8(5):119. doi: 10.3390/biomedicines8050119 (PMC7277448; doi:10.3390/biomedicines8050119)
Supplement: Supplementary file 1 [file biomedicines-08-00119-s001.pdf]

Supplementary Materials

Discovery of hordenine as a potential inhibitor of pyruvate dehydrogenase kinase 3: Implication in lung cancer therapy

Saleha Anwar[1], Taj Mohammad[1], Anas Shamsi[1], Aarfa Queen[2], Shahnaz Parveen[3,4], Suaib Luqman[3], Gulam Mustafa Hasan[5], Khalid A. Alamry[6], Naved Azum[6], Abdullah M. Asiri[6,7], Md. Imtaiyaz Hassan[1,*]

1 Centre for Interdisciplinary Research in Basic Sciences, Jamia Millia Islamia, Jamia Nagar, New Delhi 110025, INDIA.
2 Department of Chemistry, Jamia Millia Islamia, Jamia Nagar, New Delhi 110025, INDIA.
3 Molecular Bioprospection Department, CSIR-Central Institute of Medicinal and Aromatic Plants, Lucknow-226015, Uttar Pradesh, India.
4 Academy of Scientific and Innovative Research (AcSIR), Ghaziabad-201002, Uttar Pradesh, India.
5 Department of Biochemistry, College of Medicine, Prince Sattam Bin Abdulaziz University, P.O. Box 173, Al-Kharj – 11942, Kingdom of Saudi Arabia.
6 Chemistry Department, Faculty of Science, King Abdulaziz University, P.O. Box 80203, Jeddah 21589, Saudi Arabia.
7 Center of Excellence for Advanced Materials Research (CEAMR), King Abdulaziz University, P.O. Box 80203, Jeddah 21589, Saudi Arabia.

**To whom all correspondence should be addressed,*

Md. Imtaiyaz Hassan, Ph.D., FRSB, FRSC.
Centre for Interdisciplinary Research in Basic Sciences
Jamia Millia Islamia, Jamia Nagar
New Delhi 110025, INDIA
E-mail: mihassan@jmi.ac.in

Table S1. Binding parameters of screened natural compounds with PDK3 obtained from molecular docking and fluorescence binding studies.

S.No.	NAME OF THE COMPOUND/LIGAND	CHEMICAL STRUCTURE	PUBCHEM cID	ΔG # (kcal/mol)	Binding constant* (K) M^{-1}
**1**	Hordenine	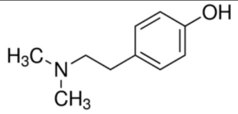	68313	–7.1	0.5 X10^{6}
2	Vincamine	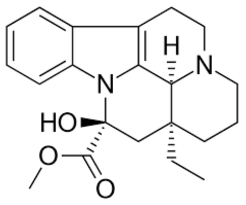	15376	–6.9	NA
3	Tryptamine	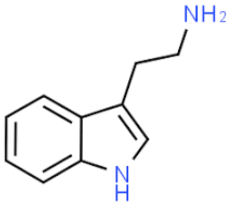	1150	–7.1	NA
4	Cinchonine	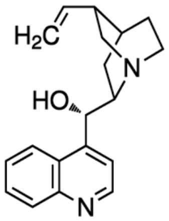	90454	–8.1	NA
5	Colcemid	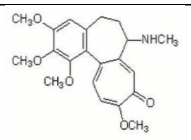	2832	–7.0	2.02 X 10^{3}

#Binding affinity of the selected compounds with PDK3 predicted through Molecular docking. *Binding constant calculated from fluorescence studies. Binding constant values could not be predicted in some cases and mentioned as not applicable (NA).
